# An Update on the Genetics of Usher Syndrome

**DOI:** 10.1155/2011/417217

**Published:** 2010-12-23

**Authors:** José M. Millán, Elena Aller, Teresa Jaijo, Fiona Blanco-Kelly, Ascensión Gimenez-Pardo, Carmen Ayuso

**Affiliations:** ^1^Unidad de Genética, Instituto de Investigación Sanitaria-La Fe, 46009 Valencia, Spain; ^2^Centro de Investigación Biomédica en Red de Enfermedades Raras (CIBERER), 46010 Valencia, Spain; ^3^Departamento de Genética Médica, Instituto de Investigación Sanitaria, Fundación Jiménez Díaz, Avenida Reyes Católicos, 2, 28040 Madrid, Spain

## Abstract

Usher syndrome (USH) is an autosomal recessive disease characterized by hearing loss, retinitis pigmentosa (RP), and, in some cases, vestibular dysfunction. It is clinically and genetically heterogeneous and is the most common cause underlying deafness and blindness of genetic origin. Clinically, USH is divided into three types. Usher type I (USH1) is the most severe form and is characterized by severe to profound congenital deafness, vestibular areflexia, and prepubertal onset of progressive RP. Type II (USH2) displays moderate to severe hearing loss, absence of vestibular dysfunction, and later onset of retinal degeneration. Type III (USH3) shows progressive postlingual hearing loss, variable onset of RP, and variable vestibular response. To date, five USH1 genes have been identified: *MYO7A* (USH1B), *CDH23* (USH1D), *PCDH15* (USH1F), *USH1C*(USH1C), and *USH1G*(USH1G). Three genes are involved in USH2, namely, *USH2A* (USH2A), *GPR98* (USH2C), and *DFNB31* (USH2D). USH3 is rare except in certain populations, and the gene responsible for this type is *USH3A*.

## 1. Introduction

Usher syndrome (USH) was first described by von Graefe in 1858 and is characterized by the association of sensorineural hearing loss, retinitis pigmentosa (RP), and, in some cases, vestibular dysfunction. Its heritability was established by Charles Usher, a British ophthalmologist [1]. The syndrome is inherited in an autosomal recessive pattern. The syndrome is the most frequent cause of deaf-blindness, accounting for more than 50% of individuals who are both deaf and blind [2, 3], about 18% of RP cases [4], and 5% of all cases of congenital deafness [5]. Its range of prevalence is 3.2–6.2/100,000 depending on the study [2, 4, 6–8].

Usher patients present progressive photoreceptor degeneration in the retina called retinitis pigmentosa, which leads to a loss of peripheral vision. This degeneration is predominantly attributable to rod dysfunction, although cones usually degenerate later in the course of the disease. Clinical symptoms may vary and include night blindness (nyctalopia) with elevated dark adaptation thresholds, abnormal electroretinogram responses, visual field constriction, abnormal retinal pigmentation including peripheral bone spicules, arterial narrowing, and optic-nerve pallor, and predisposition to myopia and posterior subcapsular cataracts [9].

The human inner ear consists of the cochlea, a snail-shaped organ which mediates sound transduction, and the vestibular labyrinth, which detects gravitational force and angular and linear accelerations. Both structures have specialized hair cells which convert mechanical stimuli into variations of intracellular potential, thus transmitting afferent nerve signals toward the brain. On the apical surface of these cells there is a mechanosensitive organelle, the hair bundle, which consists of precisely organized actin-filled projections known as stereocilia. In Usher syndrome patients, alteration in the morphogenesis and stability of stereocilia results in sensorineural hearing loss and may also cause balance defects [10].

The majority of patients with Usher syndrome usually fall into one of three clinical categories [11]. Of these, Usher syndrome type I (USH1) is the most severe form, consisting of profound hearing loss and vestibular dysfunction from birth. Moreover, onset of RP occurs earlier in USH1 than in Usher syndrome type II (USH2), which produces less severe congenital hearing loss and does not impair normal vestibular function. In most populations, USH1 accounts for approximately one-third of USH patients whereas two-thirds are classified as USH2. Usher syndrome type III (USH3) is a less common form except in such populations as Finns and Ashkenazi Jews. In this USH3 type, hearing loss is progressive and leads to variable vestibular dysfunction and onset of RP.  Table 1 outlines the clinical characteristics of each type. Some cases are not easily classifiable under the aforementioned categories and could be categorized as atypical USH syndrome [12].

All subtypes are genetically heterogeneous and 12 loci have been described, namely, *USH1B-H*, *USH2A, C-D*, and *USH3A-B* (hereditary hearing loss homepage: http://hereditaryhearingloss.org). Nine genes have been identified through the discovery of a mouse homolog or by positional cloning. There are five USH1 genes that codify known products: myosin VIIA (*MYO7A)*, the two cell-cell adhesion cadherin proteins cadherin-23 (*CDH23*) and protocadherin-15 (*PCDH15*), and the scaffold proteins harmonin (*USH1C*) and SANS (*USH1G*). The three identified USH2 genes are *USH2A*, which codes for the transmembrane protein usherin (*USH2A*); the G-protein-coupled 7-transmembrane receptor VLGR1 (*GPR98*), and whirlin (*DFNB31*), another scaffolding protein. The *USH3A* gene encodes clarin-1, which exhibits 4 transmembrane domains. Mutations in any one of these genes cause primary defects of the sensory cells in the inner ear and the photoreceptor cells of the retina, both being the source of the clinical symptoms of USH.

Many of these genes can also cause either nonsyndromic hearing loss (NSHL) or isolated RP. In fact, *MYO7A* causes DFNB2/DFNA11 [13, 14]; *USH1C* also causes DFNB18 [15, 16]; *CDH23* causes DFNB12 [17, 18]; *PCDH15* causes DFNB23 [19]; mutations in *DFNB31* also lead to DFNB31 [20, 21]. Moreover, some mutations in the *USH2A* gene cause isolated RP [22].  Table 2 shows the genetic classification of Usher syndrome, the implicated loci and responsible genes, as well as the involvement of USH in nonsyndromic hearing loss and RP.

## 2. Usher Syndrome Type I

### 2.1. Clinical Features

Usher syndrome type I is the most severe form. USH1 patients suffer from severe to profound congenital and bilateral sensorineural hearing loss. These individuals are either born completely deaf or experience hearing impairment within the first year of life and usually do not develop speech.

Constant vestibular dysfunction is present from birth; children manifest a delay in motor development and begin sitting independently and walking later than usual.

Onset of retinitis pigmentosa occurs during childhood, resulting in a progressively constricted visual field and impaired visual acuity which rapidly proceeds to blindness. Anomalies of light-evoked electrical response of the retina can be detected by electroretinography at 2-3 years of age, which allows for early diagnosis of the disease.

### 2.2. Genetic Findings

Seven loci (*USH1B–USH1H*) have been mapped and five causative Usher genes have been cloned: *MYO7A, USH1C, CDH23, PCDH15*, and *USH1G*, which are known to be implicated in USH1B, USH1C, USH1D, USH1F, and USH1G, respectively.

Several studies have investigated the *MYO7A* gene, identifying a wide range of mutations (reviewed in [23]). These reports reveal that the myosin VIIA gene bears the main responsibility for Usher type I. Its implication ranges from 29% to ~50% in different populations [24–27]. *CDH23* is probably the second most common mutated gene underlying USH1. Its prevalence accounts for 19%–35% of USH1 families [23, 25, 26, 28]. The next most frequent is *PCDH15,* reportedly involved in about 11%–19% of USH1 cases with and a significant proportion of cases due to large genomic rearrangements [25, 26, 29, 30]. The remaining genes show a minor implication in the disorder, with the *USH1C* gene accounting for 6%-7% [25, 26] and the *USH1G* for 7% as seen in USH1 populations from the United States and the United Kingdom [31]. However, in cohorts of USH1 patients from France and Spain screened for the *USH1G* gene, no pathological mutations have been identified [26, 32]. There are some exceptions to this distribution due to mutation founder effects in specific populations. As an example, the mutation c.216G>A in *USH1C* found in French Canadians of Acadian origin accounts for virtually all USH1 cases in this population [33] but has not been found in other populations; or the c.733C>T (p.R254X) in the *PCDH15* [34] gene, which is present in up to 58% of USH1 families of Ashkenazi origin.

## 3. Usher Syndrome Type II

### 3.1. Clinical Features

Firstly, RP symptoms manifest later in USH2 patients than in their USH1 counterparts, for whom onset occurs during or after puberty.

The degree of hearing impairment in patients diagnosed with USH2 increases from moderate in low frequencies to severe in high frequencies, tending to remain stable. Hearing loss is congenital but may be detected at later stages when it hinders communication. 

Vestibular function in Usher type II patients is normal.

### 3.2. Genetic Findings

To date, three loci (*USH2A, USH2C-2D*) have been proposed as being responsible for USH2, and three causative genes have been identified: *USH2A *(USH2A), *GPR98 *(USH2C), and *DFNB31* (USH2D).

Mutational screenings performed on the long isoform of the *USH2A* gene exons have shown that *USH2A* is involved in 55%–90% of USH2 cases [35–39]. Of the high number of mutations detected in this huge gene, the c.2299delG mutation is the most prevalent and accounts for 45%–15% of all mutated alleles [37, 40]. The c.2299delG mutation appears to be an ancestral mutation of European origin which spread from Europe to other regions of the world during colonization, and it shows a particular distribution decreasing in frequency from Northern to Southern Europe [40]. Again, a founder effect has been identified for the c.4338_4339delCT deletion (p.C1447QfsX29) in the *USH2A* gene which accounts for 55.6% of the USH2 alleles among Quebec French-Canadians [41].

To date, few mutation screenings have been published on *GPR98, *although based upon the results available, mutations in *GPR98* do not seem to be responsible for a large proportion of USH2 cases, approximately 3%–5.6% [39, 42].

Ebermann et al. found two *DFNB31* mutations in a German family suffering from USH2 [21]. Later, in a transnational study, Aller et al. failed to find any pathological mutation in a series of 195 USH patients [43]. *DFNB31* mutations appear to be a rare cause of recessive hearing loss and Usher syndrome.

## 4. Usher Syndrome Type III

### 4.1. Clinical Features

The onset of RP symptoms (nyctalopia, progressive constriction of visual field, and reduction of central visual acuity) is variable though usually occurs by the second decade of life.

Sensorineural hearing loss is postlingual and progressive and can appear between the first and third decade of life. In its initial stages, the degree of hearing impairment is similar to that seen in USH2, with major impairment seen in high frequencies. The progression rate is variable but, in most cases, hearing loss becomes profound. Nevertheless, hearing levels during the first stages of development are good enough to permit well-developed speech. Thus, successive audiometric examinations are needed in USH3 patients in order to obtain an accurate clinical diagnosis. The vestibular responses are also variable, with 50% of cases experiencing impairment.

### 4.2. Genetic Findings

Although the *USH3A* gene was initially described as being responsible for USH3 cases, recent studies have demonstrated that mutations in *USH3A* can also produce clinical forms of Usher that are similar to USH1 and USH2 [44, 45]. Usher syndrome type III is the least common clinical type of the syndrome in the general population. However, in some populations like the Finns or the Ashkenazi Jews, the syndrome accounts for over 40% of USH cases due to the mutation founder effect of c.300T>C (p.Y176X; known as the Finn mayor mutation) and c.143T>C (p.N48K), respectively, [46, 47].

The existence of a second locus for this clinical type (*USH3B*) was suggested by Chaïb et al. in 1997, although these findings have yet to be confirmed in [48].

## 5. The Usher Interactome

The proteins encoded by the identified USH genes belong to different protein classes. Myosin VIIA (*USH1B*) is an actin-based motor protein; harmonin (*USH1C*), SANS (*USH1G*), and whirlin (*USH2D*) are scaffolding proteins [20, 49, 50]; cadherin 23 (*USH1D*) and protocadherin 15 (*USH1F*) are cell-adhesion molecules [15, 51]; usherin (*USH2A*) and VLGR1 (*USH2C*) are transmembrane proteins with very large extracellular domains [42, 52]. Finally, clarin-1 (*USH3A*) is a protein with four transmembrane domains [53]. All these proteins have one or several protein-protein interaction domains.

USH1 and USH2 proteins are integrated in a protein network known as Usher “interactome.”

**Figure 1:**
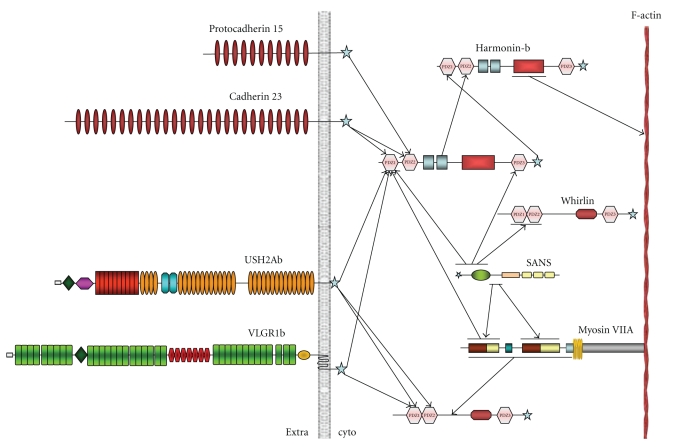
Schematic diagram illustrating the deciphered interactions within the USH protein network interactome adapted from van Wijk et al. [55].

The central core of the interactome is formed by the PDZ domain containing the homologues harmonin and whirlin and the microtubule-associated protein SANS, with the remaining USH proteins attached to this core (Figure 1).

Many of the USH proteins also interact with other proteins that are present in the inner ear and retina. These additional interacting proteins may cause Usher syndrome, nonsyndromic hearing loss, or retinal dystrophies.

Recently, one of these proteins, the protein encoded by the *PDZD7* gene, has been shown to be involved in the pathogenesis of Usher syndrome. Mutations in* PDZD7* act as negative modifiers of the phenotype [54].

The localization of the Usher proteins in the hair cells of the organ of Corti and in the photoreceptor cells suggests that they play an important role in the neurosensorial function of both the inner ear and the retina.

### 5.1. The USH Interactome in the Inner Ear

The main sites of colocalization of Usher proteins are the stereocilia and the synaptic regions of hair cells.

Usher proteins are essential for the correct development and cohesion of the hair bundle of hair cells in the cochlea and vestibular organ (reviewed in [56–58]).

In murine models, hair cells in the developing inner ear, known as stereocilia, maintain their cohesion by interstereocilia fibrous links and links with the kinocilium. There are several types of links depending on the stage of hair-cell development. In the mouse, transient lateral links appear at very early stages of stereocilia formation, but while other links appear at the base of stereocilia (ankle links), these lateral links diminish progressively throughout development. Later, ankle links diminish, and tip and horizontal links appear and are preserved in adulthood [10].

The large extracellular domains of the cell adhesion proteins cadherin-23 and protocadherin-15 and the transmembrane proteins usherin and VLGR1 are part of these links. The proteins are anchored to the intracellular scaffolding proteins harmonin and/or whirlin, which connect, via myosinVIIa and possibly other interactome proteins, to the actin core of the stereocilia [55, 56, 59, 60].

The role of the different proteins in the links probably depends on the spatiotemporal stage of the links. It has been proposed that protocadherin-15 and cadherin-23 in the tip link play an essential role in triggering the mechanotransduction cascade [61]. McGee et al. proposed that usherin and VLGR1 are expressed in the transient ankle links [62].

Usher proteins also take part in the transport of vesicles from the cuticular plate to the growing apical tip of stereocilia [56].

Besides this, the presence of many of these proteins in the synaptic regions of inner and/or outer hair cells suggests that the Usher interactome might play a role in the neurotransmission of the mechanotransduction signal [58, 60, 63].

### 5.2. The USH Interactome in the Retina

There is evidence that myosin VIIa plays a role in the transport of opsin from the inner segment to the outer segment of the photoreceptors through the connecting cilium. Such evidence appears in studies in shaker-1, the mouse model defective for myosin VIIA, since shaker-1 accumulates opsin in the ciliary plasma membrane of photoreceptor cells [64, 65].

Further studies have proven that both USH1 and USH2 proteins interact in the ciliary/periciliary region of cone and rod photoreceptors. The proteins usherin, VLGR1b, and SANS are associated with the periciliary ridge complex, which is thought to be the docking side for cargo loaded post-Golgi vesicles [66]. In mammals, this specialized domain extends over the plasma membrane of the proximal part of the calycal process, which is connected via extracellular fibrous links to the plasma membrane of the connecting cilium. In the extracellular space between the membranes of the inner segment and the connecting cilium, the extracellular domains of usherin and VLGR1b may be part of these links, perhaps by means of homomeric, heteromeric, or both interactions together. Furthermore, the short intracellular domains of usherin and VLGR1b anchor to whirlin in the cytoplasm. Finally, whirlin would link to SANS and myosin VIIa, which directly interact with the cytoskeleton microtubules and F-actin filaments [67]. Cadherin-23, vezatin, and maybe other partners of the multiprotein complex that bind myosin VIIa may serve as anchors for this molecular motor at the periciliary membrane (reviewed in [57, 68]). Thus, the Usher protein network should provide mechanical support to the membrane junction between the inner segment and the connecting cilium, participating in the control of vesicle docking and cargo handover in the periciliary ridge.

Usher proteins also localize in the photoreceptor synapse, as they do in the hair cells in the organ of Corti, where they could form a complex involved in the trafficking of the synaptic vesicles [57]. However, some researchers do not support this idea since there are no mouse models with photoreceptor synaptic dysfunction [69].

In the retinal pigment epithelium (RPE) the absence of myosin VIIa causes a significant decrease in phagocytosis of outer segment disks by the pigment epithelial cells [70], suggesting a role for myosin VIIa in the shedding and phagocytosis of the distal outer segment disks by the RPE. A role involving the intracellular transport of melanosomes in the RPE cells has also been proposed for myosin VIIa [56]. The same authors suggested that protocadherin-15, together with cadherin-23 or other cadherins, could ensure proper alignment of outer segment disks of photoreceptors and apical microvilli of RPE cells through interactions with harmonin. However, none of the USH2 proteins have been shown to be present in the RPE.

Most of the USH genes are responsible not only for Usher syndrome but also for nonsyndromic hearing loss. To date, however, only one gene *(USH2A)* is known to be responsible for isolated RP, which suggests that usherin plays a main role for the photoreceptor or that the rest of the Usher proteins are not essential in the photoreceptor function.

## 6. Conclusion

### 6.1. Diagnosis

Usher syndrome is a clinically and genetically heterogeneous disorder which is important from a public health viewpoint because of the social isolation which Usher patients must endure. The first step towards correct diagnosis is proper differential diagnosis of the syndrome.

Initially, USH manifests as a sensorineural hearing impairment, sometimes with vestibular dysfunction, with RO onset occurring later in life. Several syndromes may exhibit clinical signs which are similar to USH. Differential diagnosis should take into account the presence of endocrine abnormalities such insulin resistance, type 2 diabetes, hypertriglyceridemia, hepatic dysfunction, and/or renal failure, all of them would indicate Alström syndrome or the presence of obesity, mental retardation or cognitive impairment, and postaxial polydactly and hypogenitalism, which may be indicators of a Bardet-Biedl syndrome (BBS). If a family history of X-linked inheritance is observed, or if signs of dystonia or ataxia are detected, Mohr-Tranebjaerg syndrome should be suspected.

Genetic tests could be a very powerful tool in differential diagnosis of USH patients. However, there are many factors that make the genetic study of this disease a complicated difficult one. As explained in this paper, the genes identified to date do not explain all the USH cases (this is true for BBS and Alström syndromes as well), and the variable nature of the proteins involved in USH and the complexity of the USH interactome make identifying novel genes a difficult task. This is due to genetic and allelic heterogeneity, which contribute to the low rate of mutation detection, together with the possible presence of large deletions, mutations in noncoding regions, or isoforms in low concentration only present in the affected tissues. Moreover, other complex inheritance forms could modify the phenotype and its expression, as recently shown by Ebermann et al. [54]. All of these factors make the use of traditional techniques for mutation detection difficult.

Application of new technologies based on DNA chips could solve this problem; in fact, the recent creation of a specific microchip for this disease [71, 72] permits the identification of mutations in 30%–50% of the affected patients and requires only a very small DNA sample, and the technique is both cheap and fast [71, 72]. Advances in massive sequencing technologies will certainly change the approaches to molecular diagnosis of Usher syndrome.

Gene characterization and mutation screening will unravel the functional aspects and allow a phenotype-genotype correlation to be established.

### 6.2. Therapy

Currently, there is no treatment available for Usher syndrome. The hearing-loss problem can be solved by the use of hearing aids and cochlear implantation, but the retinal problem remains unsolved. Therapeutic strategies to treat retinal degeneration target the specific genetic disorder (gene therapy), slowing or stopping photoreceptor degeneration or apoptosis (e.g., growth factors or calcium blocker applications, vitamin supplementation, and endogenous cone viability factors) or even the replacement of lost cells (e.g, transplantation, use of stem or precursor cells) (reviewed in [73]). However, before these strategies can be applied to humans, animal models, pre clinical studies, and appropriately designed human clinical trials are needed to test different treatments and provide information on their safety and efficacy.

## Figures and Tables

**Table 1:** Clinical features of Usher syndrome types.

	USH1	USH2	USH3
Hearing loss	Severe to profound	Moderate to severe	Moderate to severe
	Congenital	Congenital	Progressive
	Stable	Stable	

Vestibular function	Altered	Normal	Variable
RP onset	Usually prepubertal	Around pubertyor postpubertal	Around puberty or postpubertal
Language	Unintelligible	Intelligible	Intelligible

**Table 2:** Genetic classification of Usher syndrome.

Locus	Location	Gene/protein	Function
*USH1B/DFNB2/DFNA1*	11q13.5	*MYO7A*/myosin VIIA	IE and R: transport
*USH1C/DFNB18*	11p15.1	*USH1C/*harmonin	IE and R: scaffolding
*USH1D/DFNB12*	10q22.1	*CDH23*/cadherin 23	IE: tip link formation; R: periciliary maintenance
*USH1E*	21q21	−/−	Unknown
*USH1F/DFNB23*	10q21.1	*PCDH15*/protocadherin 15	IE: tip link formation; R: periciliary maintenance
*USH1G*	17q25.1	*USH1G*/SANS	IE and R: scaffolding and protein trafficking
*USH1H*	15q22-23	*−/−*	Unknown
*USH2A*/RP	1q41	*USH2A*/usherin	IE: ankle links formation and cochlear development; R: periciliary maintenance
*USH2C*	5q14.3	*GPR98*/VLGR1	IE: ankle links formation Cochlear development; R: periciliary maintenance
*USH2D/DFNB31*	9q32-34	*DFNB31*/whirlin	IE: scaffolding and cochlear development; R: scaffolding
*USH3A*	3q25.1	*USH3A*/clarin-1	IE and R: probable role in synapsis transport*
*USH3B*	20q	*−/−*	Unknown

USH: usher syndrome; DFNB: autosomal recessive deafness; DFNA: autosomal dominant deafness; RP: retinitis pigmentosa; IE: inner ear; R: retina.

*A role in the retinal and inner ear synapses as been proposed for all the USH proteins. This remains to be elucidated.
